# Extensive Injury during Pregnancy

**Published:** 1856-11

**Authors:** 


*Extensive Injury During Pregnancy.*

In this city, last winter, a robust German female, about twenty-six years of age, and five months pregnant, fell into a well and descended fifty-one feet! She suffered an oblique fracture of the thigh, complete dislocation at the knee-joint, and a fracture both of the tibia and fibula just above the ankle! At no time, after the accident, did she manifest any signs of abortion, but went her full time, and was delivered, some time in June last, of a well-formed healthy child. It may not prove uninteresting to mention, that, during the pregnancy, the fracture in the vicinity of the ankle-joint failed to unite. After delivery, the process of reparation commenced, although slowly, and she is now regaining the use of her limb.—*Dr. H. Tyler Smith's Obstetric Lectures in London Lancet.*

